# Pregabalin for chronic cough due to lung cancer: randomized, double-blind, placebo-controlled trial

**DOI:** 10.1038/s41416-024-02913-2

**Published:** 2024-11-26

**Authors:** Vanita Noronha, Nandini Menon, Vijay M. Patil, Minit Shah, Amit Joshi, Srushti Shah, Kavita Nawale, Rohan Surve, Gunj Bafna, Shweta Jogdhankar, Priyanka Shelar, Ankush Shetake, Ashish Singh, Sushmita Salian, Pundlik Jadhav, Hetakshi Shah, Neha Mer, Ananya Vohra, Swaratika Majumdar, Shripad Banavali, Rajendra Badwe, Kumar Prabhash

**Affiliations:** 1 Department of Medical Oncology, Tata Memorial Centre, Homi Bhabha National Institute (HBNI), Mumbai, India; 2 Department of Medical Oncology, P D Hinduja Hospital & Medical Research Centre, Khar & Mahim, Mumbai, India; 3 Department of Medical Oncology, Sunrise Oncology Centre, Mumbai, India; 4 Department of Leukodystrophy Center (Neurology), Children’s Hospital of Philadelphia, Pennsylvania, PA USA; 5 Consultant Medical Oncologist, Mazumdar Shaw Medical Centre, Bengaluru, India

**Keywords:** Non-small-cell lung cancer, Non-small-cell lung cancer

## Abstract

**Background:**

Developing effective therapies for cough in lung cancer is an unmet need Neuromodulators like pregabalin may act centrally as cough suppressants.

**Methods:**

Randomized double-blind placebo-controlled study in patients with locally advanced/metastatic lung cancer and at least 2 weeks of moderate or severe cough. Randomization was 1:1 to pregabalin 300 mg orally daily or matching placebo, both administered for 9 weeks. Primary endpoint was the change in cough severity as measured by the difference in VAS scores.

**Results:**

Between Jul 2022 and Dec 2023, we enrolled 166 patients: 83 to each arm. Baseline cough severity was grade 2 in 128 (77.1%) and grade 3 in 38 (22.9%) patients; median cough duration was 12 weeks (IQR, 6–20). Systemic cancer-directed therapy was started in 78 (94.0%) and 72 (86.7%) patients in the pregabalin and placebo arms, respectively; *P* = 0.187. The mean (SD) VAS score (in mm) decreased from 71.58 (14.99) at baseline, to 45.54 (26.60) on day 7, and 22.27 (24.20) by week 9 in the pregabalin arm; and 71.75 (17.58), 46.35 (25.00), and 23.08 (22.42), respectively in the placebo arm; *P* = 0.877.

**Conclusion:**

Pregabalin does not significantly decrease cough in patients with lung cancer. Systemic cancer-directed therapy is the most effective antitussive.

**Clinical trial registration:**

Name of the registry: Clinical Trials Registry India Registration number: CTRI/2020/11/029275 Website: www.ctri.nic.in

## Introduction

Cough develops in 55–90% of patients with lung cancer [1–3]. Harle et al. studied the prevalence of cough and its impact in 202 consecutive patients with lung cancer. They found that, depending on the tool used, the prevalence of cough ranged from 57% (by the visual analog scale [VAS]) to 67% (by the Manchester Cough in Lung Cancer [MCLCS] scale). The mean cough severity was 32 mm as measured on a 100 mm VAS (IQR = 20–51), and the impact was moderate as measured on the MCLCS (mean score = 22, IQR = 16–27). As per the MCLCS, 39% patients reported moderate to severe cough, 18% patients reported substantial distress due to cough, and 15% experienced significant sleep disruption due to cough. Overall, 50% of patients desired therapy for cough, and 25% found their cough to be painful. The only factor that correlated with less cough was active cancer-directed therapy; 40% of patients on therapy reported cough as compared to 54% patients who were not on therapy, *P* = 0.04 [4]. Multiple studies have found that cough detracts from the quality of life (QoL) in patients with lung cancer [3, 5]. An exploratory study by Molassiotis et al. revealed that cough caused embarrassment, psychological distress, and difficulty socializing in patients with lung cancer [6]. With recent exciting advances in the management of lung cancer, including immune checkpoint inhibitors, targeted therapies, antibody-drug conjugates and other innovative treatment strategies, patients with advanced lung cancer are living longer, and it is imperative that we appropriately manage patients’ symptoms that detract from QoL. There has been limited research on the management of cough in patients with lung cancer. There are no official guidelines for managing cancer-related cough from any of the official oncology societies or organizations, including the American Society of Clinical Oncology (ASCO), European Society of Medical Oncology (ESMO), Multinational Association of Supportive Care in Cancer (MASCC), or the National Comprehensive Cancer Network (NCCN). The most recent American College of Chest Physicians (CHEST) guidelines from 2017 for the symptomatic treatment of cough in patients with lung cancer were based on 17 trials of low-quality evidence. The guidelines stated that conducting high-quality research studies on this topic was the need of the hour [7].

One of the mechanisms of central cough suppressant antitussives is to increase the activation threshold of the central neurons in the brainstem. As pain and cough share similar pathways, anticonvulsants like gabapentin and pregabalin which are traditionally used for treating neuropathic pain, have been explored as cough treatments. These drugs may exert their antitussive effect by centrally inhibiting the cough reflex. They bind and inhibit the α2δ subunit of the presynaptic calcium channels, inhibiting glutamate release into the central synapse, similar to the mechanism of glutamate receptor antagonists like dextromethorphan [8]. Several studies have evaluated the role of gabapentin as an antitussive in chronic cough, including two small randomized controlled trials in patients with refractory cough that had lasted for at least 8 weeks, without active infection, pulmonary pathology or any structural disease (*n* = 56; *n* = 62) [9, 10]. A systematic review of the clinical data of the use of gabapentin in chronic cough by Shi et al. in 2018 (which included the two randomized trials, two retrospective case series, two prospective case series and one case report) reported that gabapentin resulted in improvement in cough severity and cough-related QoL, with an overall 68% improvement in cough. They concluded that gabapentin was more efficacious and had an acceptable safety profile than a placebo or standard cough medicines [11]. However, all the patients included in the systematic review had idiopathic refractory chronic cough; lung cancer or any other identifiable etiology of the cough like infection or structural lung disease were exclusion criteria. Thus, the efficacy and safety of gabapentin for chronic cough in patients with lung cancer is unknown. Pregabalin has also been evaluated in patients with chronic cough, although not in patients with lung cancer. In a randomized trial in 40 patients with chronic cough, pregabalin added to speech pathology therapy, resulted in significantly improved perceived cough severity, improved cough-related QoL, and reduced cough sensitivity, as compared to placebo with speech pathology therapy [12]. In a systematic review on drug therapies for chronic refractory cough, Ryan et al. concluded that gabapentin and pregabalin led to a reduction in the cough frequency and improved the QoL [13].

Given the importance of managing chronic cough in patients with lung cancer, the paucity of evidence-based therapeutic strategies, and the data on the efficacy of gabapentin and pregabalin for refractory cough in patients without lung cancer, we conducted a randomized study to evaluate the role of pregabalin as an antitussive in patients with lung cancer and chronic cough.

## Patients and methods

### General study details

This was a phase III randomized, double-blind, placebo-controlled trial conducted in the Outpatient Department of Medical Oncology at the Tata Memorial Hospital, a tertiary oncology-only academic hospital in Mumbai, India. The study protocol was approved by the Institutional Ethics Committee and the study was monitored by the Data Safety and Monitoring Subcommittee (Study protocol provided as Supplementary Appendix 1). The protocol was written and implemented by the authors. All authors had access to the data and contributed to the manuscript development, including writing the first draft, revising, and approving the final version. The authors vouch for the completeness and accuracy of the data and adherence to the protocol. The study was prospectively registered with the Clinical Trials Registry-India, CTRI/2020/11/029275 (www.ctri.nic.in). All patients provided written informed consent. We strictly adhered to the ethical guidelines as established by the Declaration of Helsinki, and Good Clinical Practice Guidelines. Funding was obtained from the Tata Memorial Centre Research Administration Council, and placebo capsules were provided by ACG Associated Capsules Pvt Ltd. The funding agencies had no role in the planning or implementation of the study, data analysis or decision to publish the manuscript.

### Aims/Objectives

Our aim was to evaluate the efficacy and safety of pregabalin, as compared to placebo, for treating chronic cough in patients with lung cancer. The primary endpoint was the change in the cough severity as measured by the difference in the VAS score from baseline to week 9 between patients treated with pregabalin 300 mg orally daily, compared to matching placebo. Secondary endpoints included an assessment of the change in cough impact from baseline to day 7, and to week 9 using the MCLCS between patients treated with pregabalin 300 mg orally daily and matching placebo; the change in cough severity as assessed by VAS from baseline to day 7, between patients treated with pregabalin 300 mg orally daily and matching placebo; adverse effects; and QoL as measured by the European Organization for Research and Treatment of Cancer (EORTC) QLQ-C30 and the lung cancer specific module LC13.

### Eligibility criteria

We enrolled patients who were 18 years or older with locally advanced or metastatic non-small cell lung cancer, and an Eastern Cooperative Oncology Group (ECOG) performance status (PS) between 0 and 2. The ECOG PS is a measure of the patient’s functionality, particularly in terms of their ability to care for themselves, do work, and perform physical activities. It is scored from 0 to 5, where 0 indicates that the patient is fully functional and carrying out all activities without any restrictions, 1 indicates that the patient is ambulatory, can carry out light work like basic housework and office work, but is unable to perform physically strenuous work, and 2 indicates a patient who is ambulatory, can carry out all self-care activities, is up and about more than half the waking hours, but is unable to do any work [14]. Patients had to have either moderate (grade 2) or severe (grade 3) cough lasting for at least 2 weeks. The traditional definition of chronic cough is cough that has lasted for at least 8 weeks [15]. However, this definition of chronic cough is relevant mainly for non-malignant causes of cough. In patients with cough due to lung cancer, we felt the need for a revised definition, given the patient’s limited life expectancy. Since cough lasting for 2 or more weeks requires radiological and laboratory evaluation for an underlying pathological condition namely, tuberculosis and lung cancer, we decided to adopt this as our definition for chronic cough in lung cancer for the purpose of this study. We found this a clinically relevant definition which facilitated timely evaluation and diagnosis. The grading of the cough severity was as per the Common Terminology Criteria for Adverse Events (CTCAE), version 5 [16], in which grade 2 cough signified moderate symptoms, requiring medical intervention, and limiting instrumental activities of daily living; and grade 3 cough signified severe symptoms limiting self-care activities. Patients had to have a creatinine clearance (calculated by the Cockcroft-Gault method) of ≥60 mL/min and had to be willing and able to limit to one alcoholic beverage per day. We excluded patients who had earlier been treated with pregabalin or gabapentin, had hypersensitivity to pregabalin or gabapentin, and those who were pregnant or breast-feeding at the time of screening.

### Study methodology

Baseline laboratory tests (complete hemogram, liver and renal function tests, serum electrolytes) were performed, and patients who fulfilled the eligibility criteria underwent randomization. Randomization was carried out centrally by an independent biostatistician at the Clinical Research Secretariat of the Tata Memorial Hospital. Participants were randomly assigned in a 1:1 ratio to pregabalin or placebo group. A permuted-block randomization sequence was created using a variable block size of 2 and 4, using the RALLOC a Stata Module in Stata, version 16. The randomization was emailed to the unblinded member of the study team, who dispensed the appropriate blinded study medication according to the allocated arm. All other members of the study team, patients and caregivers were blinded to which medicine the patient was receiving. Patients randomized to pregabalin were started on 75 mg orally daily, with dose escalation over the next 7 days to a maximum dose of 300 mg orally daily, continued for 9 weeks, followed by dose de-escalation over 7 days, and then discontinued. Patients randomized to the placebo arm were started on the matching placebo according to the same schedule, i.e., dose escalation over 7 days, then continued orally daily for 9 weeks, followed by de-escalation over 1 week, and then discontinued. Patients were evaluated at baseline, day 7, and week 9. Evaluation included an assessment of symptoms, adverse events (graded as per CTCAE version 5), concomitant medications including any additional antitussives, cough severity by VAS, and cough impact by MCLCS. We chose to evaluate cough with VAS and MCLCS as both these questionnaires have been validated in patients with lung cancer [17], as opposed to other commonly used questionnaires like the Leicester Cough Questionnaire (LCQ) [18], which has not been validated in chronic cough due to lung cancer. CTCAE is a grading system established by the National Cancer Institute (USA) to describe the severity of adverse events. In general, grade 1 adverse events are mild, associated with no or mild symptoms, are clinical or diagnostic observations only, and do not necessitate interventions; grade 2 adverse events are moderate, limit instrumental activities of daily living, and require minimal, local or non-invasive interventions; grade 3 are severe but not immediately life-threatening adverse events, are disabling and limit self-care activities of daily living, and require hospitalization or prolongation of hospitalization; grade 4 are life-threatening adverse events and require urgent intervention; grade 5 are fatal adverse events [16]. Dose holds, and dose modifications were done according to the protocol. For patients in either arm whose cough was not controlled by the study drug, additional antitussive drugs were prescribed in a stepwise fashion, with expectorants/mucolytics (guaifenesin/bromhexine) added first, followed by antihistamines (diphenhydramine/chlorpheniramine), antiadrenergics (phenylephrine/terbutaline), and narcotics (codeine, dextromethorphan).

The decision regarding cancer-directed therapy was made by the treating oncologist-the regimen administered was recorded. QoL was assessed by the EORTC QLQ-C30 (a general QoL 30-item questionnaire to assess various general aspects that impact the QoL in patients with cancer) [19] and the lung cancer specific module, LC13 [20], which is a 13-item lung-cancer specific questionnaire that includes lung cancer-associated symptoms (i.e., coughing, hemoptysis, dyspnea and pain) and side-effects from conventional chemo- and radiotherapy (i.e., hair loss, neuropathy, sore mouth and dysphagia).

### Cough assessment

Patients were asked on day 7 and at week 9 whether they had any improvement in cough (Yes/No) and what percentage change from baseline they had experienced in their cough. We used the VAS to assess the cough severity. This is a straight 10 cm line, marked 0 at one end (labelled “No cough”) and 100 mm at the other end (labelled, “Worst cough ever”). The patient was asked to mark the point on the line that best correlated with the perceived severity of his/her cough. A higher score indicated worse cough severity. The use of VAS has been validated in patients with chronic cough, and a decrease of >30 mm in VAS was correlated with a clinically meaningful cough reduction [21]. The cough impact was evaluated through the MCLCS: a form comprising 10 questions that describe the patient’s cough experience in the preceding week; each question has five possible answers scored as 1 [never] to 5 [all the time]; total score ranges from 10 to 50; high score indicates worse cough impact [22]. Both VAS and MCLCS were filled out by the patients, with help from trained research coordinators and social workers.

### Sample size

The primary endpoint, i.e., the difference in the VAS from baseline to 9 weeks was used to determine the sample size. We assumed a medium effect size of 0.5, type 1 error of 5%, and power of 80%. Using a two-tailed t-test, and equal 1:1 allocation to the two arms, the estimated sample size was 128. As a large proportion of the patients recruited would have advanced lung cancer, we accounted for a lost-to-follow-up rate (i.e., patients for whom the 9-week VAS score would not be obtainable) of approximately 30%. The final estimated sample size was 166.

### Statistical analysis

Analysis was performed using the Statistical Package for the Social Sciences (SPSS) software (IBM SPSS Statistics for Windows, Version 23.0. Armonk, NY: IBM Corp.), R Studio version 2024.04.0 + 735 and Python for Statistical Computing. Descriptive statistics were used to summarize the baseline characteristics including demographic data, diagnosis and treatment details, and adverse events, using absolute numbers and simple percentages. The association of demographic characteristics and adverse events with the treatment groups was assessed using Chi-square test or Fisher’s test.

Analysis was performed according to the modified intention-to-treat principle, because the primary end point required each patient to have completed the subjective cough evaluation by VAS at baseline and at week 9. All patients with a cough severity assessment at baseline and at 9 weeks were included for analysis of the primary efficacy endpoint. Patients for whom cough assessment was available at baseline and day 7 were included in the analysis of the secondary efficacy endpoints. We used the complete-case analysis to handle missing data, i.e., missing observations were excluded from the analysis. The normality of the change in scores from baseline to day 7, and baseline to week 9 on the EORTC QLQ-C30 and LC13, MCLCS and VAS scales was assessed using Kolmogorov-Smirnov’s test for normality. The distribution of non-normally distributed change in scores between the two treatment groups was compared using Mann–Whitney U test. The non-normally distributed change in scores between the two groups was summarized using mean (standard deviation [SD]). The changes in scores from baseline to day 7 and to week 9 have been graphically presented using the mean (95% confidence interval [CI]) plot.

To measure cough severity, the VAS score at 9 weeks was subtracted from the score at baseline. The means of the change from the baseline scores were calculated for all patients in each arm and the means were compared between the two arms using independent samples Mann–Whitney U test. A two-sided *P*-value of less than 0.05 was considered statistically significant. The effect size was determined using Cohen’s d statistic, which is a measure of the difference between two means divided by an estimate of a pooled standard deviation. As per conventional classification, an effect size of 0.2 was considered small, 0.5 moderate, and 0.8 large.

QoL data were scored as per the procedure described in the EORTC scoring manual. Different subdomain wise QoL data were generated from the QoL dataset. A subdomain wise comparison was performed between the arms, using the linear mixed effect model. We handled the missing QoL values by taking the average value of the column and replacing the missing data.

## Results

**Fig. 1:**
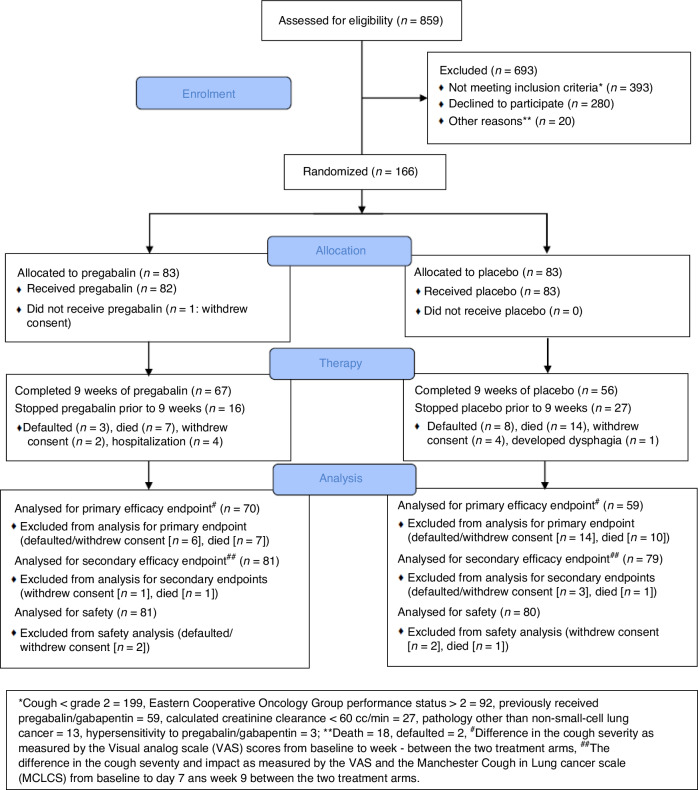
The process of screening, enrollment, randomization, treatment, and analysis of the patients in the cough study.

Between July 2022 and Dec 2023, we randomized 166 patients: 83 to each arm. The details of patient screening, enrollment, random allocation, therapy, and analysis are provided in Fig. 1. Baseline demographic, clinical, and disease-related information are provided in Table 1 and Supplementary Appendix 2. The prevalence of chronic obstructive pulmonary disease (COPD) and asthma was low (6.6%) for a population of patients with lung cancer. There are two possible explanations for this. One is underdiagnosis prior to the presentation of lung cancer. However, at our institution, pulmonary medicine physicians form an integral part of the lung cancer multidisciplinary team, and patients are evaluated and prescribed medicines for COPD, if required, following the diagnosis of lung cancer [23]. The second possible explanation for the low prevalence of COPD in our cohort of patients with lung cancer is that the majority of patients with lung cancer at our institution (52%) are never-smokers [1, 24].

**Table 1:** Baseline demographic, clinical, disease-related and cough-related information for the patients enrolled in the cough study

Characteristic	All patients, in number (%) (*n* = 166)	Pregabalin arm, in number (%) (*n* = 83)	Placebo arm, in number (%) (*n* = 83)	*P*-value
Age, in years				
Median (IQR)	56 (47–63)	56 (48–63)	56 (46–62)	0.889
≥60 years	60 (36.1)	29 (34.9)	31 (37.3)	0.747
Sex				
Male	112 (67.5)	58 (69.9)	54 (65.1)	0.508
Female	54 (32.5)	25 (30.1)	29 (34.9)	
Tobacco use				
None	68 (41.0)	29 (34.9)	39 (47.0)	0.114
Smoking	65 (39.2)	32 (38.6)	33 (39.8)	0.874
Smokeless	43 (25.9)	28 (33.7)	15 (18.1)	0.021
Comorbidities				
None	80 (48.2)	45 (54.2)	35 (42.2)	0.120
Present	86 (51.8)	38 (45.8)	48 (57.8)	
ECOG performance status^a^				
1	143 (86.1)	70 (84.3)	73 (88.0)	0.500
2	23 (13.9)	13 (15.7)	10 (12.0)	
Extent of disease				
Locally advanced	11 (6.6)	7 (8.4)	4 (4.8)	0.535
Metastatic	155 (93.4)	76 (91.6)	79 (95.2)	
Histopathology				
Adenocarcinoma	121 (72.9)	63 (75.9)	58 (69.9)	0.729
Squamous	28 (16.9)	12 (14.5)	16 (19.3)	
Other^b^	17 (10.2)	8 (19.6)	74 (10.8)	
Molecular test report				
No driver alteration	71 (42.8)	38 (45.8)	33 (39.8)	0.290
*EGFR*	32 (19.3)	17 (20.5)	15 (18.1)	
*ALK*	25 (15.1)	11 (13.3)	14 (16.9)	
*ERBB2*	3 (1.8)	3 (3.6)	0	
Other^c^	24 (13.8)	11 (13.2)	13 (14.4)	
Molecular testing not done	12 (7.2)	3 (3.6)	9 (10.8)	
Intent of cancer-directed therapy				
Palliative	159 (95.8)	79 (95.2)	80 (96.4)	1.000
Curative	7 (4.2)	4 (4.8)	3 (3.6)	
Dyspnea severity at baseline				
Grade 0	69 (41.6)	34 (41.0)	35 (42.2)	0.763
Grade 1	68 (41.0)	34 (41.0)	34 (41.0)	
Grade 2	28 (16.9)	13 (15.7)	15 (18.1)	
Grade 3	1 (0.6)	0	1 (1.2)	
Cough severity at baseline				
Grade 2	128 (77.1)	66 (79.5)	62 (74.7)	0.460
Grade 3	38 (22.9)	17 (20.5)	21 (25.3)	
Cough duration at presentation, in weeks				
Median (IQR)	12 (6–20)	12 (7–20)	12 (4–20)	0.897
Range	2–104	2–79	2–104	
Number of therapies given for cough, at presentation				
Median (IQR)	1 (1–1)	1 (1–1)	1 (1–1)	0.684
Range	0–3	0–3	1–3	
Cancer-directed therapy started				
Yes	150 (90.4)	78 (94.0)	72 (86.7)	0.187
No	16 (9.6)	5 (6.0)	11 (13.3)	

*IQR* interquartile range, *COPD* chronic obstructive pulmonary disease, *ECOG* Eastern Cooperative Oncology Group, *EGFR* epidermal growth factor receptor, *ALK* anaplastic lymphoma kinase, *ERBB2* v-erb-b2 avian erythroblastic leukemia viral oncogene homologue 2 [HER2].

^a^The ECOG PS is a measure of the patient’s functionality, particularly in terms of their ability to care for themselves, do work, and their physical activities. It is scored from 0 to 5, where 0 indicates that the patient is fully functional and carrying out all activities without any restrictions, 1 indicates that the patient is ambulatory, can carry out light work like basic housework and office work, but is unable to perform physically strenuous work, and 2 indicates a patient who is ambulatory, can carry out all selfcare activities, is up and about more than half the waking hours, but is unable to do any work.

^b^Other histopathologies- large cell neuroendocrine [2 (2.4), 1 (1.20)], non-small cell lung carcinoma, not otherwise specified [3 (3.6), 5 (6.0)], poorly differentiated carcinoma [2 (2.4), 2 (2.4)], adenosquamous [0, 1 (1.2)], carcinoma with hepatoid differentiation [1 (1.2), 0] in pregabalin and placebo arms, respectively.

^c^Other molecular test reports – *ROS1* [0, 2 (2.4)], *MET* [0, 1 (1.2)], *EGFR* exon 20 insertion [2 (2.4), 2 (2.4)], *EGFR* sensitizing mutation +*ALK* fusion [1 (1.2), 0], *BRAF* [3 (3.6), 1 (1.2)], *KRAS* [5 (6.60), 6 (7.2)] in pregabalin and placebo arms, respectively.

**Fig. 2:**
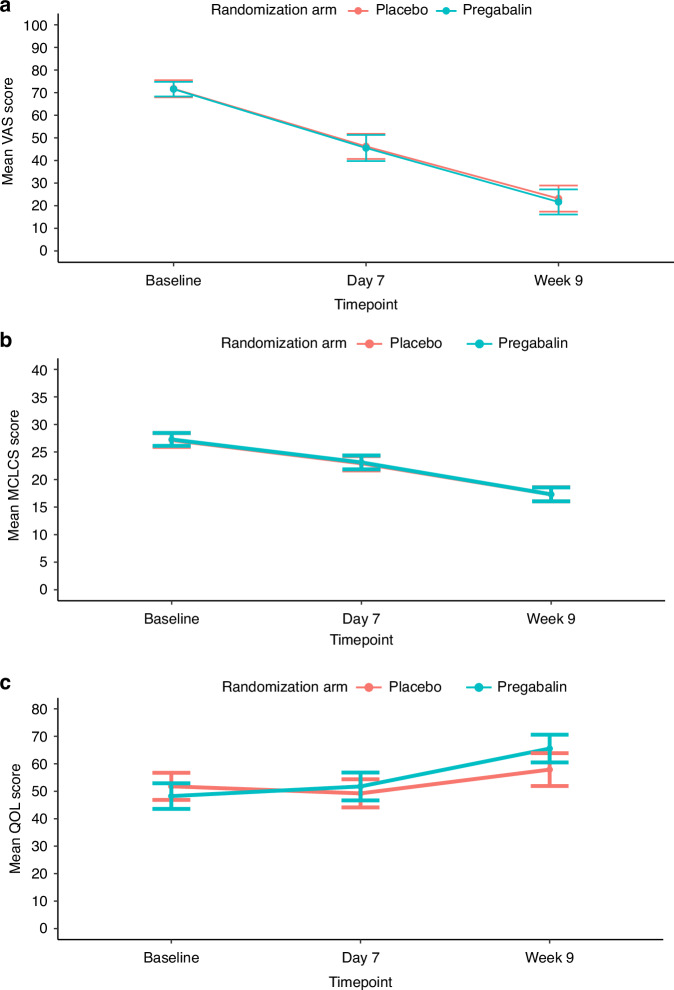

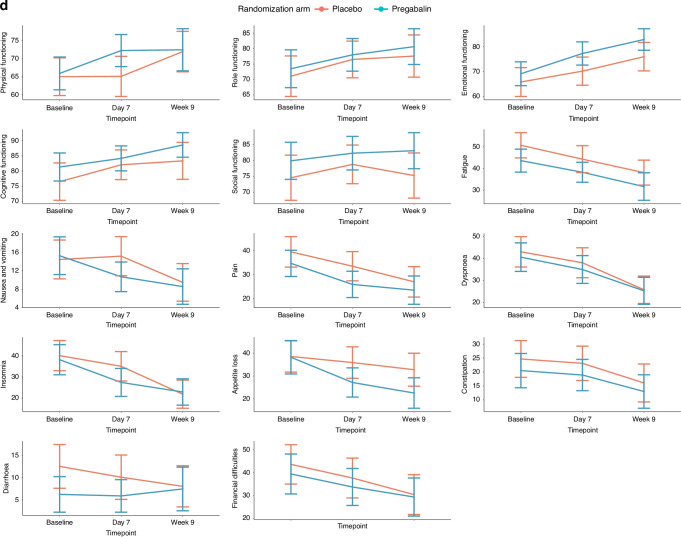

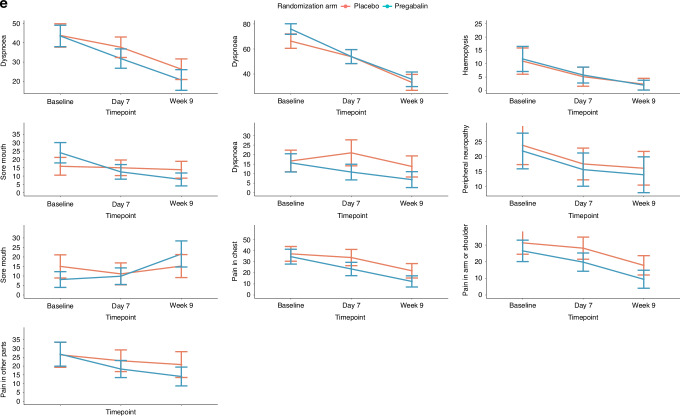
Graphical representation of the change in cough and the quality of life (QoL) from baseline to day 7 and to week 9 in patients with lung cancer and chronic cough treated with pregabalin or placebo in the cough study. For all plots, the blue line denotes the pregabalin arm [Arm A], and the red line denotes the placebo arm [Arm B]. **a** The change in cough severity as measured by the visual analog scale (VAS); **b** the change in cough impact as measured by the Manchester Cough in Lung Cancer Scale (MCLCS); **c** the change in the QoL as measured by the European Organization for Research and Treatment of Cancer (EORTC) QLQ-C30 (Global health status); **d** the change in QoL as measured by the EORTC QLQ-C30 (Functional scales and Symptom scales/items); and e the change in QoL as measured by the EORTC lung cancer specific module (LC13).

Symptomatic relief in cough was reported by 118 (71.1%) patients on day 7, and by 92 (55.4%) by week 9. Reported cough relief was similar between the two arms (Table 2). This was also reflected by the objective measures used for cough severity (VAS) and cough impact (MCLCS) (Supplementary Appendix 3). The mean difference in the VAS score at week 9 from that at baseline was similar between the two arms: −41.91 (SD, 26.92) in the pregabalin arm, and −48.20 (SD, 24.60) in the placebo arm, *P* = 0.877, effect size = 0.03. Similarly, there was no significant difference in the VAS scores on day 7 as compared to baseline between the two arms, and between the MCLCS scores on day 7 or at week 9 between the two arms. The QoL scores were also similar between the two arms at the various timepoints (Fig. 2, Supplementary Appendix 3).

**Table 2:** Cough details, and other therapies, including cancer-directed therapy in patients with lung cancer and chronic cough, treated with pregabalin or placebo as part of the cough study

Cough and therapy details	All patients, in number (%) (*n* = 166)	Pregabalin arm, in number (%) (*n* = 83)	Placebo arm, in number (%) (*n* = 83)	*P*-value
Cough symptom relief on day 7				
Yes	118 (71.1)	56 (67.5)	62 (74.7)	0.191
No	35 (21.1)	22 (26.5)	13 (15.7)	
Cough relief in % on day 7, in median (IQR)	20 (0–50)	10 (0–50)	20 (10–50)	0.431
Cough grade on day 7				
No cough	2 (1.3)	2 (2.5)	0	0.552
Grade 1	77 (48.1)	39 (48.1)	38 (48.1)	
Grade 2	72 (45.0)	36 (44.4)	36 (45.6)	
Grade 3	9 (5.6)	4 (4.9)	5 (6.3)	
Not assessed				
Cough symptom relief at week 9				
Yes	92 (55.4)	50 (66.2)	42 (50.6)	0.230
No	8 (4.8)	2 (2.4)	6 (7.2)	
Cough relief in % at week 9, in median (IQR)	80 (50–100)	80 (50–100)	70 (23.5–92.5)	0.182
Cough grade at week 9				
No cough	33 (19.9)	20 (24.1)	13 (15.7)	0.129
Grade 1	79 (47.6)	42 (50.6)	37 (44.6)	
Grade 2	10 (6.0)	6 (7.2)	4 (4.8)	
Grade 3	1 (0.6)	1 (1.2)	0	
Not assessed	43 (25.9)	15 (18.1)	28 (33.7)	
Requirement for additional medicines for cough while on study				
No	84 (50.6)	45 (54.2)	39 (47.0)	0.362
Yes	81 (48.8)	37 (44.6)	44 (53.0)	
Additional medicines while on study				
Inhalers	34 (34.5)	17 (34.5)	17 (34.4)	0.622
Steroids	37 (22.9)	21 (25.3)	17 (20.5)	0.465
Morphine	32 (19.3)	15 (18.1)	17 (20.5)	0.694
Etoricoxib	7 (4.2)	4 (4.8)	3 (3.6)	1.00
Tramadol	81 (48.8)	40 (48.2)	41 (49.4)	0.877
Antibiotics	70 (42.2)	36 (43.3)	34 (41.0)	0.535

*IQR* interquartile range.

Approximately half of the enrolled patients required additional antitussive medicines while on the study; this requirement for additional cough medicine was not significantly different between the two arms. Approximately 20% of the patients received steroids, and 20% received morphine while on study, which may have impacted patients’ cough; this requirement for steroids, morphine, and other medicines that may potentially have acted on the cough was similar between the two arms. Systemic cancer-directed therapy was started in 90% of the patients, 78 (94%) in the pregabalin arm and 72 (86.7%) in the placebo arm, *P* = 0.187. The common systemic therapy regimens were pemetrexed + platinum (with or without immunotherapy/bevacizumab/Mycidac-C) in 31%, EGFR tyrosine kinase inhibitor in 17%, and paclitaxel + platinum in 15% (Table 2).

The dose of the study medicine was reduced for only one (1.2%) patient in the placebo arm, due to ataxia. The study medicine was held in 6 (7.3%) patients in the pregabalin arm and 3 (3.6%) in the placebo arm; in all patients this was because of hospitalization for an acute medical event. Pregabalin did not result in a significant increase in any grade or grade ≥3 toxicities (Table 3). Any grade toxicities occurred in 80 (98.8%) and 80 (100%) patients in the pregabalin and placebo arms, respectively, *P* = 0.650. Grade 3 and higher adverse events occurred in 34 (42%) and 34 (41.3%) patients in the pregabalin and placebo arms, respectively, *P* = 0.926.

**Table 3:** Adverse events of patients enroled in the cough study, both in the pregabalin arm and the placebo arm

Characteristic	Pregabalin arm, in number (%) (*n* = 81)				Placebo arm, in number (%) (*n* = 80)				*P*-value
	Grade 1	Grade 2	Grade 3	Grade 4	Grade 1	Grade 2	Grade 3	Grade 4	
Hypertension	17 (21.0)	15 (18.5)	1 (1.2)	0	17 (21.0)	15 (18.5)	1 (1.2)	0	0.355
Weight loss	7 (8.4)	3 (3.6)	0	0	6 (7.2)	1 (1.2)	0	0	0.728
Dizziness	27 (33.3)	1 (1.2)	0	0	23 (28.7)	0	0	0	0.482
Somnolence	21 (25.9)	5 (6.2)	0	0	21 (26.3)	0	0	0	0.077
Constipation	32 (39.5)	3 (3.7)	0	0	25 (31.3)	1 (1.3)	0	0	0.287
Xerostomia	23 (28.4)	0	0	0	26 (32.5)	0	0	0	0.571
Increased appetite	13 (15.7)	1 (1.2)	0	0	12 (14.5)	0	0	0	0.742
Headache	23 (28.2)	1 (1.2)	0	0	24 (30.0)	0	0	0	0.599
Vomiting	16 (19.8)	2 (2.5)	0	0	17 (21.3)	2 (2.5)	0	0	0.972
Mucositis	4 (4.9)	1 (1.2)	0	0	7 (8.8)	3 (3.8)	0	0	0.357
Fatigue	30 (37.0)	13 (16.0)	4 (4.9)	0	20 (25.0)	15 (18.8)	2 (2.5)	0	0.278
Diarrhea	7 (8.6)	2 (2.5)	4 (4.9)	0	9 (11.3)	5 (6.3)	0	0	0.135
Anorexia	10 (12.3)	4 (4.9)	0	0	15 (18.8)	2 (2.5)	0		0.410
Anemia	32 (39.5)	25 (30.9)	8 (9.9)	1 (1.2)	39 (48.8)	17 (21.3)	9 (11.3)	1 (1.3)	0.681
Thrombocytopenia	16 (19.8)	5 (6.02)	3 (3.7)	0	14 (17.5)	4 (5.0)	2 (2.5)	2 (2.5)	0.654
Elevated hepatic transaminases	28(34.6)	5 (6.2)	2 (2.5)	1 (1.2)	21 (26.3)	6 (7.5)	1 (1.3)	1 (1.3)	0.774
Hyponatremia	32 (39.5)	14 (17.3)	13 (16.0)	0	28 (35.0)	12 (15.0)	15 (18.8)	2 (2.5)	0.630
Hypokalemia	15 (18.5)	2 (2.5)	2 (2.5)	0	11 (13.8)	0	0	0	0.173

Adverse events were noted, regardless of attribution, and were graded according to the Common Terminology Criteria for Adverse Events, version 5 [16].

## Discussion

Cough is a distressing symptom that detracts from both physical and emotional well-being and is almost universally present in patients with lung cancer. Developing effective therapies for chronic cough in patients with lung cancer is an urgent unmet need, with no well-designed trials addressing this issue, and consequently, no official guidelines issued by any of the major oncology societies. To bridge this gap, we conducted a double-blind, placebo-controlled study evaluating the efficacy of pregabalin in patients with chronic cough in the setting of lung cancer. We found that all patients enrolled in the study had progressive improvement in cough from baseline to the end of study at week 9, and this cough relief was similar between the patients treated with pregabalin and those who received placebo. Thus, pregabalin did not appear to be more effective than a placebo in relieving cough in patients with lung cancer. Over 90% of the patients were started on systemic anticancer therapies while on the study, and this factor probably contributed to the relief in cough in both the arms. This was also reflected by the fact that patients in both arms had an improvement in their QoL throughout the course of the study. Thus, systemic cancer-directed therapy is a highly effective antitussive in patients with lung cancer and remains the treatment of choice for the management of chronic cough in patients with lung cancer.

Pregabalin is a neuromodulator indicated for the treatment of neuropathic pain, postherpetic neuralgia, partial seizures, and anxiety disorders [25]. Several investigators have reported that pregabalin prescribed for other reasons like postherpetic neuralgia led to cough improvement [26–28]. Vertigan et al. reported the only randomized study that established the efficacy of pregabalin in patients with chronic cough [12]. They randomized 40 patients with chronic refractory cough to undergo speech pathology therapy with or without pregabalin to a maximum dose of 300 mg orally daily for 14 weeks. Pregabalin resulted in an improvement in cough as measured by the LCQ (mean difference 3.5, 95% CI, 1.1–5.8), cough VAS (mean difference 25.1, 95% CI, 10.6–39.6), improvement in capsaicin-cough sensitivity, but no decrease in cough frequency. Saint-Pierre studied 50 consecutive patients who had been prescribed pregabalin for chronic cough and found that 56% patients had an improvement of at least 1.3 (the minimal clinically important difference) in the LCQ score [29]. Contrary to the results of these studies, we found that pregabalin did not lead to a significant reduction in cough in patients with lung cancer.

In addition to the fact that almost all patients enrolled in our study received cancer-directed therapy that may have masked any potential effect of pregabalin on cough, the other possible explanations for the observed lack of antitussive effect of pregabalin include alternative pathways for malignant cough that pregabalin does not act on, potentially sub-therapeutic dosing of pregabalin, or suboptimal timing of cough assessment. Based on our clinical experience with the use of pregabalin for neuropathy and earlier studies on the use of pregabalin for cough, we capped the dose of pregabalin at 300 mg daily. However, the maximum recommended dose of pregabalin for fibromyalgia is 450 mg daily, and that for partial seizures and neuropathic pain from spinal cord injuries is 600 mg daily [30]. Pregabalin exhibits a dose-response relationship in diabetic peripheral neuropathy and post-herpetic neuralgia, with higher efficacy from 600 mg as compared to 300 mg or 150 mg [31, 32]. It is plausible that the antitussive effect of pregabalin in patients with lung cancer may be exerted only at doses higher than 300 mg daily. The timing of cough assessment is also crucial. In our earlier study evaluating the role of aprepitant for cough in patients with lung cancer, the primary endpoint was cough severity on day 9 as measured by VAS [33]. However, pregabalin takes time to exert maximal effect, and the majority of studies evaluating pregabalin for neuropathy have used endpoints between 5 and 13 weeks [34]. In the randomized study that established the role of pregabalin for radiotherapy-related neuropathic pain in head and neck cancer, the primary endpoint was pain reduction at 16 weeks [35]. In our study, we therefore, planned to assess the improvement in cough at 9 weeks, which would allow sufficient time for pregabalin to exert its maximal effect, as well as would be convenient for patients, who would conventionally undergo repeat evaluation and staging 2–3 months after starting systemic cancer-directed therapy. However, this timing of the cough assessment also allowed for the antitussive effect of cancer-directed therapy, perhaps masking any potential antitussive effect of pregabalin.

In patients with lung cancer, the pathophysiology of chronic cough is complex and multifactorial, and involves multiple pathways and mechanisms, including chronic activation by inflammatory cytokines that act as nociceptors causing peripheral sensitization of the afferent vagal neuronal pathways, central hypersensitivity with hyperactivity of the brainstem cough receptors, and downregulation of endogenous cough inhibitory pathways [36–38]. It is likely that effective therapy of cough in patients with lung cancer would require targeting multiple pathways, which may not be achievable with a single drug. In general, antitussive medications usually comprise a combination of more than one drug with different mechanisms of action, again emphasizing the fact that effective cough therapy requires targeting multiple pathways together. Cancer-directed therapy, by virtue of its effect on the tumor, could potentially target all the active pathways triggered by the malignancy responsible for chronic cough in lung cancer, thus making it the most effect antitussive.

In our study, 77% of patients had grade 2 cough and 23% had grade 3 cough at baseline; cough had lasted for a median of 12 weeks (range, 2–104). The median VAS at baseline was 72 (moderate cough severity), and the median MCLCS was 27 (moderate impact). Patients had received a variety of treatments for cough, most commonly chlorpheniramine in 51%, dextromethorphan in 46%, guaifenesin in 28%, codeine in 26%, triprolidine in 25%, terbutaline in 23%, and bromhexine in 22%. Despite these antitussive therapies, all our patients reported significant cough that was bothersome at the time of enrollment in the study, reiterating that cough is indeed a common symptom that impacts patients’ QoL. The current standard of care for chronic cough in lung cancer has been outlined in the 2017 CHEST guidelines [7]. Patients should undergo a thorough evaluation for reversible causes of cough and should be taught cough suppression exercises. Endobronchial brachytherapy is recommended in patients unsuitable for surgery, radiation, or chemotherapy. Pharmacologic management includes stepwise trials of demulcents, opioids, peripheral antitussives, and local anesthetics. In patients with persistent cough despite all measures, other drugs may be tried including diazepam, gabapentin, carbamazepine, baclofen, amitriptyline, or thalidomide [7]. Based on the results of our study, pregabalin and perhaps, gabapentin, are not effective and may be removed from this list. Besides the routinely used antitussives, the only other medicine that has been shown to be effective in cough in lung cancer is aprepitant [33, 39]. Thus, effective antitussive therapies need to be urgently developed for patients with lung cancer and chronic cough.

The main limitation of our study was that almost all patients were started on systemic cancer-directed therapy. To establish the antitussive effect of a new or repurposed drug for cough, the ideal time would be either while patients are awaiting the start of therapy, i.e., a window of opportunity study while performing the workup and molecular testing, or in patients who have received two to three lines of systemic cancer-directed therapy, and in whom effective cancer therapies are not available. Besides, symptom control is most challenging in patients who are not on systemic therapy, and in those for whom there is limited effective cancer therapy. The location of the disease, i.e., intrabronchial or peripheral was reported on the imaging or assessed via bronchoscopy in only a small fraction of the patients, and hence we have not analyzed this information. This could have impacted the cough presentation and severity. We capped the maximum dose of pregabalin at 300 mg a day, which may not have been sufficient for the antitussive effect. The use of other therapies like steroids and opioids may have been confounding factors. As our primary endpoint was cough severity at 9 weeks, we excluded 37 (22.3%) patients who did not undergo the 9-week assessment. This was expected as most patients had advanced lung cancer, and we had accounted for this in the sample size calculation. The adverse events noted in the study were likely to be more reflective of the systemic cancer-directed therapy, leading to some bias in the safety analysis.

### Interpretation

Pregabalin does not lead to a significant improvement in cough in patients with lung cancer. Systemic cancer-directed therapy is the most effective antitussive for chronic cough caused by lung cancer. Developing effective antitussive agents is an unmet need and well-designed studies are urgently needed.

## Supplementary information

SupplementaryAppendices_Pregabalin_edited_clean_version.docx

Related Manuscript File

## Data Availability

The individual de-identified data will be available on request to the corresponding author until 5 years after publication. Requests beyond this timeframe will be considered on a case-by-case basis.
