# Supplementary material for: Pregabalin for chronic cough due to lung cancer: randomized, double-blind, placebo-controlled trial
Source: Br J Cancer. 2024 Nov 26;132(1):58–68. doi: 10.1038/s41416-024-02913-2 (PMC11723996; doi:10.1038/s41416-024-02913-2)
Supplement: Supplementary file 1 — SupplementaryAppendices_Pregabalin_edited_clean_version.docx [file 41416_2024_2913_MOESM1_ESM.docx]

**SUPPLEMENTARY MATERIAL**

Manuscript title: ’Pregabalin for chronic cough due to lung cancer: Randomized, double-blind, placebo-controlled trial’

Short Title: **A randomized trial on pregabalin for chronic cough in lung cancer**

|  | **Name** | **Highest Degree obtained** | **Email address** | **Department** | **Institution** |
| --- | --- | --- | --- | --- | --- |
| 1 | Vanita Noronha | DM | [vanita.noronha@gmail.com](mailto:vanita.noronha@gmail.com) | Medical Oncology | Tata Memorial Hospital, Mumbai, Homi Bhabha National Institute, Mumbai, India |
| 2 | Nandini Menon | DNB | [nandini.menon1412@gmail.com](mailto:nandini.menon1412@gmail.com) | Medical Oncology | Tata Memorial Hospital, Mumbai, Homi Bhabha National Institute, Mumbai, India |
| 3 | Vijay Patil | DM | [vijaypgi@gmail.com](mailto:vijaypgi@gmail.com) | Medical Oncology | P D Hinduja Hospital & Medical Research Centre, Khar & Mahim |
| 4 | Minit Shah | DM | [minitjshah@gmail.com](mailto:minitjshah@gmail.com) | Medical Oncology | Tata Memorial Hospital, Mumbai, Homi Bhabha National Institute, Mumbai, India |
| 5 | Amit Joshi | DM | [dramitjoshi74@gmail.com](mailto:dramitjoshi74@gmail.com) | Medical Oncology | Tata Memorial Hospital, Mumbai, Homi Bhabha National Institute, Mumbai, India |
| 6 | Srushti Shah | BHMS, PDCR | [drsrushti25@gmail.com](mailto:drsrushti25@gmail.com) | Medical Oncology | Tata Memorial Hospital, Mumbai, Homi Bhabha National Institute, Mumbai, India |
| 7 | Kavita Nawale | MSc, PGDCR | [kavitatmh@gmail.com](mailto:kavitatmh@gmail.com) | Medical Oncology | Tata Memorial Hospital, Mumbai, Homi Bhabha National Institute, Mumbai, India |
| 8 | Rohan Surve | BDS, PGDMR | [rohansurve529@gmail.com](mailto:rohansurve529@gmail.com) | Medical Oncology | Tata Memorial Hospital, Mumbai, Homi Bhabha National Institute, Mumbai, India |
| 9 | Gunj Bafna | BDS, PGDACR | bafnagunj@gmail.com | Medical Oncology | Sunrise Oncology Centre |
| 10 | Shweta Jogdhankar | MSc | shwetajogdhankar1995@gmail.com | Medical Oncology | Tata Memorial Hospital, Mumbai, Homi Bhabha National Institute, Mumbai, India |
| 11 | Priyanka Shelar | B.Com | priyankapadale1987@gmail.com | Medical Oncology | Tata Memorial Hospital, Mumbai, Homi Bhabha National Institute, Mumbai, India |
| 12 | Ankush Shetake | MSc | ankya2460@gmail.com | Medical Oncology | Tata Memorial Hospital, Mumbai, Homi Bhabha National Institute, Mumbai, India |
| 13 | Ashish Singh | BAMS, MSc | ashroks143@gmail.com | Medical Oncology | Tata Memorial Hospital, Mumbai, Homi Bhabha National Institute, Mumbai, India |
| 14 | Sushmita Salian | MSc | sushi.11.salian@gmail.com | Medical Oncology | Tata Memorial Hospital, Mumbai, Homi Bhabha National Institute, Mumbai, India |
| 15 | Pundlik Jadhav | MSc | pundliknashik@gmail.com | Medical Oncology | Tata Memorial Hospital, Mumbai, Homi Bhabha National Institute, Mumbai, India |
| 16 | Hetakshi Shah | BHMS, MSc | drhetakshi@gmail.com | Medical Oncology | Tata Memorial Hospital, Mumbai, Homi Bhabha National Institute, Mumbai, India |
| 17 | Neha Mer | MSc | merneha.msccr@gmail.com | Medical Oncology | Tata Memorial Hospital, Mumbai, Homi Bhabha National Institute, Mumbai, India |
| 18 | Ananya Vohra | BSc | ananya.nv@gmail.com | Leukodystrophy Center (Neurology) | Children’s Hospital of Philadelphia |
| 19 | Swaratika Majumdar | DM | swaratika@hotmail.com | Medical Oncology | Consultant Medical Oncologist, Mazumdar Shaw Medical Centre, Bengaluru, India |
| 20 | Shripad Banavali | MD | banavali_2000@yahoo.com | Medical Oncology | Tata Memorial Hospital, Mumbai, Homi Bhabha National Institute, Mumbai, India |
| 21 | Rajendra Badwe | MS | badwera@gmail.com | Surgical Oncology | Tata Memorial Hospital, Mumbai, Homi Bhabha National Institute, Mumbai, India |
| 22 | Kumar Prabhash | DM | kumarprabhashtmh@gmail.com | Medical Oncology | Tata Memorial Hospital, Mumbai, Homi Bhabha National Institute, Mumbai, India |

SUPPLEMENTARY APPENDIX 2: Details of comorbidities, antitussive use at baseline, and cancer-directed therapy for the patients enrolled in the cough study

| **Characteristic** | **All patients, in number (%)**  **(n=166)** | **Pregabalin arm, in number (%) (n=83)** | **Placebo arm, in number (%) (n=83)** | ***P*-value** |
| --- | --- | --- | --- | --- |
| **Comorbidities** | | | | |
| Diabetes mellitus | 30 (18.1) | 14 (16.9) | 16 (19.4) | 0.687 |
| Hypertension | 44 (26.5) | 20 (24.1) | 24 (28.9) | 0.482 |
| Heart disease | 9 (5.4) | 6 (7.2) | 3 (3.6) | 0.496 |
| COPD/asthma | 11 (6.6) | 7 (8.4) | 4 (4.8) | 0.535 |
| Tuberculosis | 12 (7.2) | 4 (4.8) | 8 (9.6) | 0.370 |
| Hypothyroidism | 9 (5.4) | 3 (3.6) | 6 (7.2) | 0.496 |
| Other* | 18 (0.6) | 7 (1.2) | 11 (1.5) | 0.318 |
| **Antitussive use at presentation** | | | | |
| Chlorpheniramine | 85 (51.2) | 38 (45.8) | 47 (56.6) | 0.162 |
| Dextromethorphan | 77 (46.4) | 36 (43.4) | 41 (49.4) | 0.436 |
| Guaifenesin | 47 (28.3) | 26 (31.3) | 21 (25.3) | 0.389 |
| Codeine | 43 (25.9) | 20 (24.1) | 23 (27.7) | 0.595 |
| Triprolidine | 41 (24.7) | 20 (24.1) | 21 (25.3) | 0.857 |
| Terbutaline | 38 (22.9) | 21 (25.3) | 17 (20.5) | 0.460 |
| Bromhexine | 37 (22.3) | 21 (25.3) | 16 (19.3) | 0.351 |
| Sodium citrate | 10 (6.0) | 4 (4.8) | 6 (7.2) | 0.746 |
| Phenylephrine | 9 (5.4) | 4 (4.8) | 5 (6.0) | 1.000 |
| Salbutamol/levosalbutamol | 7 (4.2) | 4 (4.8) | 3 (3.6) | 1.000 |
| Diphenhydramine | 7 (4.2) | 4 (4.8) | 3 (3.6) | 1.000 |
| Ambroxol | 6 (3.6) | 3 (3.6) | 3 (3.6) | 1.000 |
| Ammonium chloride | 6 (3.6) | 3 (3.6) | 3 (3.6) | 1.000 |
| Noscapine | 3 (1.8) | 0 | 3 (3.6) | 0.245 |
| Acetylcysteine | 1 (0.6) | 1 (1.2) | 0 | 1.000 |
| Loratidine | 1 (0.6) | 1 (1.2) | 0 | 1.000 |
| Levocloperastine | 1 (0.6) | 0 | 1 (1.2) | 1.000 |
| Acebrophylline | 1 (0.6) | 1 (1.2) | 0 | 1.000 |
| Benzonatate | 1 (0.6) | 1 (1.2) | 0 | 1.000 |
| Unknown antitussive (ayurvedic, over-the-counter antitussive) | 7 (4.2) | 3 (3.6) | 4 (4.8) | 0.699 |
| **Details of systemic cancer-directed therapy** | | | | |
| Pemetrexed + platinum +/- immunotherapy/Mycidac-C/bevacizumab | 51 (30.7) | 27 (32.5) | 24 (28.9) | 0.389 |
| Paclitaxel + platinum | 24 (14.5) | 12 (14.5) | 12 (14.5) |  |
| Etoposide + platinum | 7 (4.2) | 4 (4.8) | 3 (3.6) |  |
| Taxane (paclitaxel/docetaxel) +/- ramucirumab | 8 (4.8) | 3 (3.6) | 5 (6.0) |  |
| Gemcitabine + afatinib | 1 (0.6) | 1 (1.2) | 0 |  |
| Platinum (single agent carboplatin) | 6 (3.6) | 5 (6.0) | 1 (1.2) |  |
| Immunotherapy (nivolumab) | 5 (3.0) | 1 (1.2) | 4 (4.8) |  |
| EGFR TKI + pemetrexed + carboplatin | 8 (4.8) | 6 (7.2) | 2 (2.4) |  |
| EGFR TKI (gefitinib/erlotinib/osimertinib) | 28 (16.9) | 12 (14.5) | 16 (19.3) |  |
| ALK TKI (crizotinib/ceritinib) | 13 (7.8) | 7 (8.4) | 6 (7.2) |  |

ACRONYMS: EGFR=epidermal growth factor receptor, TKI=tyrosine kinase receptor, ALK=anaplastic lymphoma kinase

*Other comorbidities - alcoholic liver disease [1 (1.2), 0], appendix surgery [1 (1.2), 0], benign prostatic hypertrophy [1 (1.2), 0], cataract surgery [1 (1.2), 0], frontal lobe lipoma [1 (1.2), 0], hysterectomy for excessive discharge [1 (1.2), 0], left eye blindness [1 (1.20) , 0], bilateral optic neuritis [0, 1 (1.2)], carcinoma tongue [0, 1 (1.2)], gout [0, 1 (1.2), hepatitis B [0, 2 (2.4), hepatitis C [0,1 (1.2)], HIV [0, 1 (1.2)], hyperlipidemia [0, 2 (2.4)], obstructive cholelithiasis, gastroesophageal reflux disease [0,1 (1.2)], stroke [0,1 (1.2)] in pregabalin and placebo arms, respectively.

SUPPLEMENTARY APPENDIX 3: Details of cough severity as measured by the visual analog scale (VAS), cough impact as measured by the Manchester Cough in Lung Cancer Scale (MCLCS), and quality of life as measured by the European Organization for Research and Treatment of Cancer (EORTC) QLQ-C30 and the lung cancer specific module LC13, in patients with lung cancer and chronic cough treated with pregabalin or placebo in the cough study

| **Type of questionnaire/domain** | **Scores of patients in pregabalin arm, in mean (SD)** | | | **Scores of patients in the placebo arm, in mean (SD)** | | | **P- value**  **(Baseline to day 7)** | **P-value**  **(Baseline to week 9)** | **Effect size (Baseline to day 7)** | **Effect size (Baseline to week 9)** |
| --- | --- | --- | --- | --- | --- | --- | --- | --- | --- | --- |
|  | **Baseline** | **Day 7** | **Week 9** | **Baseline** | **Day 7** | **Week 9** |  |  |  |  |
| VAS | 71.58 (14.99) | 45.54 (26.60) | 22.27 (24.20) | 71.75  (17.58) | 46.35 (25.00) | 23.08  (22.42) | 0.906 | 0.877 | 0.018 | 0.027 |
| MCLCS | 27.28 (5.44) | 23.14 (5.79) | 17.46 (5.36) | 27.16  (5.83) | 22.97  (5.88) | 17.33  (5.03) | 0.886 | 0.906 | 0.002 | 0.020 |
| **Quality of life** | | | | | | | | | | |
| Global QoL | 48.23 (21.61) | 51.75 (23.34) | 64.50 (22.17) | 51.79  (23.02) | 49.01  (23.13) | 58.04  (23.19) | 0.088 | 0.068 | 0.279 | 0.334 |
| **QUALITY OF LIFE FUNCTIONAL SCALES** | | | | | | | | | | |
| Physical functioning | 65.94 (20.99) | 72.24 (20.27) | 71.14 (25.60) | 65.04  (24.15) | 65.14  (24.89) | 72.20  (21.73) | 0.106 | 0.941 | 0.257 | 0.013 |
| Role functioning | 73.49 (28.40) | 77.98 (24.27) | 79.29 (25.61) | 71.08  (30.47) | 76.79  (26.88) | 77.97  (26.70) | 0.870 | 0.562 | 0.025 | 0.103 |
| Emotional functioning | 69.14 (22.28) | 77.30 (21.41) | 81.72 (19.97) | 65.83  (27.02) | 70.36  (25.50) | 76.41  (22.49) | 0.384 | 0.449 | 0.138 | 0.134 |
| Cognitive functioning | 81.33 (21.37) | 84.16 (18.80) | 87.92 (17.36) | 76.51  (28.52) | 81.86  (22.04) | 83.62  (23.67 | 0.527 | 0.583 | 0.099 | 0.096 |
| Social functioning | 79.92 (27.14) | 82.31 (24.20) | 82.14 (25.90) | 74.60  (33.05) | 79.06  (27.45) | 75.71  (27.74) | 0.765 | 0.693 | 0.047 | 0.069 |
| **QoL symptom scales** | | | | | | | | | | |
| Fatigue | 43.63  (24.50) | 38.27  (21.15) | 33.17  (28.15) | 50.67  (26.94) | 44.16  (27.96) | 37.85  (22.33) | 0.664 | 0.727 | 0.069 | 0.062 |
| Nausea and vomiting | 15.26  (18.98) | 10.70  (14.74) | 9.29  (16.70) | 14.46  (19.62) | 15.40  (19.20) | 9.32  (15.86) | 0.058 | 0.403 | 0.301 | 0.149 |
| Pain | 34.74  (25.53) | 25.93  (25.77) | 25.48  (27.47) | 39.56  (29.64) | 33.75  (27.60) | 26.55  (24.97) | 0.850 | 0.510 | 0.026 | 0.117 |
| Dyspnea | 40.56  (29.93) | (34.98  (28.82) | 26.57  (26.56) | 42.97  (31.89) | 38.40  (330.70) | 25.42  (24.24) | 0.952 | 0.763 | 0.009 | 0.053 |
| Insomnia | 38.21  (33.7) | 27.43  (30.55) | 24.15  (27.35) | 40.16  (33.25) | 35.06  (31.48) | 21.47  (26.09) | 0.336 | 0.939 | 0.155 | 0.013 |
| Appetite loss | 38.21  (34.00) | 27.16  (29.40) | 23.33  (28.57) | 24.69  (30.63) | 23.42  (27.76) | 15.82  (26.52) | 0.078 | 0.152 | 0.281 | 0.255 |
| Constipation | 20.51  (28.54) | 18.93  (25.79) | 12.63  (25.33) | 24.69  (30.63) | 23.25  (27.77) | 15.82  (26.52) | 0.675 | 0.662 | 0.068 | 0.079 |
| Diarrhea | 6.25  (18.44) | 5.90  (16.67) | 7.25  (20.51) | 12.5  (22.74) | 9.96  (22.34) | 7.91  (17.87) | 0.479 | 0.251 | 0.115 | 0.209 |
| Financial difficulties | 39.43  (40.62) | 33.74  (37.08) | 28.92  (35.46) | 43.62  (40.01) | 37.18  (39.47 | 29.89  (34.02) | 0.965 | 0.513 | 0.007 | 0.119 |
| **Lung cancer specific QOL (LC13) symptom scales** | | | | | | | | | | |
| Dyspnea | 43.50  (25.75) | 31.82  (23.02) | 21.43  (22.96) | 43.77  (28.16) | 38.05  (24.17) | 25.89  (20.93) | 0.193 | 0.175 | 0.206 | 0.240 |
| Cough | 75.90  (19.70) | 53.91  (25.59) | 36.23  (24.75) | 66.27  (26.80) | 53.59  (25.27) | 33.33  (24.76) | 0.049 | 0.104 | 0.313 | 0.287 |
| Hemoptysis | 11.78  (21.83) | 5.76  (13.73) | 1.90  (7.79) | 10.98  (22.86) | 5.06  (16.09) | 2.29  (8.52) | 0.776 | 0.738 | 0.045 | 0.060 |
| Sore mouth | 23.98  (28.34) | 12.5  (20.11) | 8.21  (16.57) | 15.83  (24.69) | 15.19  (21.21) | 13.55  (19.68) | 0.043 | 0.016 | 0.325 | 0.439 |
| Dysphagia | 15.66 (22.29) | 10.83  (18.96) | 7.62  (18.10) | 16.67  (26.34) | 21.10  (30.74) | 13.56  (21.55) | 0.013 | 0.205 | 0.400 | 0.225 |
| Peripheral neuropathy | 21.81  (27.47) | 15.64  (25.32) | 14.01  (25.18) | 23.69  (29.69) | 17.30  (23.79) | 15.82  (21.76) | 0.689 | 0.955 | 0.063 | 0.010 |
| Alopecia | 8.13  (19.29) | 9.88  (20.03) | 20.95  (29.03) | 15.04  (28.28) | 11.39  (26.09) | 14.94  (23.51) | 0.072 | 0.034 | 0.288 | 0.386 |
| Pain in the chest | 34.54  (31.00) | 23.46  (27.61) | 12.86  (21.46) | 37.08  (30.92) | 33.76  (32.89) | 21.46  (25.34) | 0.289 | 0.643 | 0.169 | 0.082 |
| Pain in the arm or shoulder | 26.51  (29.80) | 19.75  (25.13) | 10.14  (23.08) | 31.33  (31.81) | 28.20  (29.95) | 17.51  (22.62) | 0.878 | 0.934 | 0.024 | 0.014 |
| Pain in other parts | 26.75  (31.75) | 18.30  (22.39) | 15.66  (24.96) | 26.41  (33.04) | 23.61  (28.22) | 20.47  (28.70) | 0.177 | 0.614 | 0.234 | 0.094 |

ACRONYMS: VAS=Visual Analog Scale, MCLCS=Manchester Cough in Lung Cancer Scale, QoL=Quality of life, SD=Standard Deviation
