# Supplementary material for: Pregabalin for chronic cough due to lung cancer: randomized, double-blind, placebo-controlled trial
Source: Br J Cancer. 2024 Nov 26;132(1):58–68. doi: 10.1038/s41416-024-02913-2 (PMC11723996; doi:10.1038/s41416-024-02913-2)
Supplement: Supplementary file 2 — Related Manuscript File [file 41416_2024_2913_MOESM2_ESM.docx]

# TITLE: **Pregabalin for chronic cough in patients with lung cancer: A randomized double-blind placebo-controlled trial.**

**Authors:** Kumar Prabhash, Vanita Noronha, Swaratika Majumdar

**Document type:** Clinical Research Protocol

**Financial Disclosure:** None

**Version:** 4.0

**Date:** 23^rd^ Jan 2023

**Prior versions and dates:** 1.0 (July 4, 2019), 2.0 (July 3, 2020), 3.0 (Oct 12, 2020)

| Principal Investigators: | 1. Dr. Vanita Noronha   Professor, Medical Oncology, Tata Memorial Hospital, Parel, Mumbai  Email: [vanita.noronha@gmail.com](mailto:vanita.noronha@gmail.com)  Ph: 022-24177031   1. Dr. Kumar Prabhash   Professor and Head of Department, Medical Oncology, Tata Memorial Hospital, Parel, Mumbai  Email: [kprabhash1@gmail.com](mailto:kprabhash1@gmail.com)  Ph: 022-24177214 |
| --- | --- |
| Co-Investigators: | 1. Dr Amit Joshi   Professor, Medical Oncology, Tata Memorial Hospital, Parel, Mumbai  Email: dramit74@yahoo.com  Ph: 022-24177031   1. Dr Srushti Jain   Trial coordinator, Medical Oncology, Tata Memorial Hospital, Parel, Mumbai  Email: drsrushti25@gmail.com  Ph: 022-24177000   1. Dr. Nandini Menon   Assistant Professor, Department of Medical Oncology, Parel, Mumbai-400012  Ph: 022-24177000   1. Dr. Ajaykumar Singh   Assistant Professor, Department of Medical Oncology, Parel, Mumbai-400012  Ph: 022-24177000 |

# 2. PROTOCOL SYNOPSIS

**Study Title:** Pregabalin for chronic cough in patients with lung cancer: a randomized double-blind placebo-controlled trial.

**Aim**: To evaluate the efficacy and safety of pregabalin in improving the severity of cough in patients with lung cancer compared to placebo.

**Primary Objective:** To study the effect of pregabalin (maximal tolerated dose of 300 mg per day) as compared to placebo on cough severity as measured by the Visual Analog Scale (VAS) between baseline and 9 weeks of treatment.

**Secondary Objectives:**

- To study the effect of pregabalin (maximal tolerated dose of 300 mg per day) as compared to placebo on cough severity as measured by the Manchester Cough in Lung Cancer Scale (MCLCS) score between baseline and 9 weeks of treatment.
- To study the change in VAS and MCLCS scores between baseline and day 7 of therapy with pregabalin (maximal tolerable dose of 300 mg per day) as compared to matching placebo.
- To study the effect of pregabalin therapy as compared to placebo on improvement in quality of life (QoL) in lung cancer patients with chronic cough.
- To describe the adverse effects of pregabalin in lung cancer patients with chronic cough.

**Study Population**

Patients with suspected/diagnosed advanced lung cancer presenting with cough lasting for 2 or more weeks and planned for palliative intent therapy.

**Sample Size:** 166

**Treatment Plan:**

Patients with advanced lung cancer complaining of cough lasting for 2 or more weeks will be randomized to receive either pregabalin (at maximal tolerated dose of 300 mg orally daily) (Arm A) or a placebo (Arm B).

Severity of cough will be assessed using Visual Analog Scale (VAS) and Manchester Cough in Lung Cancer Scale (MCLCS). QoL will be assessed using the EORTC QLQ C30 and LC13. VAS, MCLCS and QoL forms will be administered at the time of enrolment in the study, on day 7, and at the end of study visit (9 weeks). Toxicity data will be collected at every visit.

**Duration of Study:** 24 Months

# 1. INTRODUCTION

Cough is a distressing symptom afflicting nearly 90% of patients with lung cancer(1). Adequate cough suppression in lung cancer patients is an unmet clinical need and requires strategies beyond current medical practice. Development of effective antitussives is hampered by our lack of understanding of complex cough pathways and objective tools for assessment of cough severity. Advances in cancer management have led to improved survival of patients with incurable lung cancer thereby making palliation of chronic cough necessary in clinical care.(2)

Although the epidemiological definition of chronic cough is cough that has lasted for three or more months, the current clinical guidelines use an arbitrary cut-off of eight weeks (3). This definition of chronic cough is applicable for non-malignant causes of cough. In patients with cough due to lung cancer, there is a need for a revised definition, given the patient’s limited life expectancy. Since cough lasting for two or more weeks requires radiological and laboratory evaluation for an underlying pathological condition namely, tuberculosis and lung cancer, we have decided to adopt this as our definition for chronic cough in lung cancer for the purpose of this study. We find this a clinically relevant definition which facilitates timely evaluation and diagnosis.

Although only 2% of these patients are likely to have lung cancer, nearly 65% of patients with cancer have cough at presentation (4). Understanding the central and peripheral mechanisms of cough in lung cancer is imperative to finding effective antitussive strategies. The cough reflex is fundamentally a defensive mechanism. Peripheral pathways comprise of Aδ fibers carrying fast conducting stimuli from larger airways and C fibers from small airways which relay slow conducting, inhibitory cough signal via the vagus nerve. These communicate with central cough pathways via two neurotransmitters, neurokinin A and glutamate. (5) Albeit the central efferent cough center is located in the medulla oblongata, it is subject to modulation by supramedullary and pain pathways.

Chronic cough can be considered akin to neuropathic pain. It is likely that opiates and neuromodulatory drugs like gabapentin and pregabalin exert an antitussive effect by influencing pain pathways (6). Cough in lung cancer can be related to a large central tumor stimulating Aδ fibers or interstitial lesions damaging the inhibitory C fibers (5).

The three domains of cough severity are its intensity, frequency and disruptiveness. When evaluating any drug for efficacy against cough, it is important to use validated tools to assess cough severity and the impact of cough on quality of life (QoL), so that the efficacy of various drugs or interventions can be compared between studies. The Visual Analog Scale (VAS) is a simple and reproducible subjective tool to assess cough severity. The VAS is a 10 cm line, marked 0 at one end and 100 mm at the other end. The end marked 0 is labelled as ‘No cough’ and the end labelled as 100 mm is marked ‘worst cough ever’. The patient is asked to mark the point on the line that best correlates with his/her cough in terms of his/her perceived severity. A higher score indicates worse cough severity. VAS has been described and validated in patients with lung cancer (16,17). A seven-item Cough Severity Diary is another subjective tool evaluating all three domains of cough [disruptive (two items), frequency (three items) and intensity (two items)] (7). Objective evaluation of cough frequency can be performed by ambulatory cough monitoring (ACM). This involves the patient strapping on a recording device with a miniature microphone for up to 24 hours. Its interpretation is complex and remains unvalidated in patients with cough related to lung cancer. Manchester Cough in Lung Cancer Scale (MCLCS) was one of the earliest tools to be extensively studied in lung cancer patients. It assesses the severity, frequency, distress and the impact of cough. MCLCS is a questionnaire consisting of ten questions that describe the patient’s cough experience in the preceding week; each question has five possible answers scored as one (never) to five (all the time); the score ranges from one to fifty; higher score indicates worse cough impact. In addition, The Leicester cough questionnaire (LCQ) and the cough-specific quality of life questionnaire (CQLQ) are also useful health status questionnaires for adults with chronic cough. EORTC Q30 and the lung cancer specific LC13 questionnaire have been used to evaluate global QoL in lung cancer patients. However, a validated tool evaluating cough specific QoL in lung cancer patients is lacking (8).

Lack of randomized trials, heterogenous patient population and minimal use of valid cough assessment tools have led to the largely empirical management of cough in patients with lung cancer. It was acknowledged by Molassiotis *et al*. that the evidence for the use of all cough suppressants is at best based on good clinical practice (9). There is no set standard of care with respect to the choice and dosage of antitussive agents. In 2015, a Cochrane Systematic Review concluded that no practice recommendations could be made given that all the studies evaluating the pharmacological therapy of cough had significant bias (10). Clinical Expert Guidelines for the Management of Cough in Lung Cancer (2010) suggested that opioids (hydrocodone, dihydrocodeine, dextromethorphan, morphine) were better than sodium cromoglycate, and benzonatate in curbing cough in lung cancer patients (9). Of the various opioids, dextromethorphan curtailed cough the best (HR, 0.37; 95% CI, 0.19-0.56; P=0.0008) (11). Local anesthetic agents (nebulized lidocaine and benzonatate) were additional options. Failing these, experimental options such as aprepitant, baclofen, thalidomide, gabapentin/pregabalin, carbamazepine or amitriptyline could be considered. Providing a pragmatic approach to dealing with cough suppression, Molassiotis *et al*. conceptualized a ‘cough pyramid’ which was akin to the ‘pain ladder’(9). Cancer directed therapy (chemotherapy/external beam radiation therapy) remains the most effective strategy for controlling cough in patients with lung cancer. New experimental drugs, including pregabalin and gabapentin, form the apex of the cough pyramid; these require further exploration.

Neuromodulatory drugs like gabapentin and pregabalin have been successfully tried in neuropathic pain. Their utility in cough suppression too has been studied in small randomized trials though none of the trials involved patients with lung cancer. A study in 2012 compared gabapentin to placebo for chronic cough. 32 patients randomized to the gabapentin arm were found to have improved cough specific QoL (difference in LCQ score of 1.8; P=0.004) (12). At the dose used (maximum dose of 1800 mg/day), 30% of the patients treated with gabapentin had significant side-effects notably fatigue and nausea, as compared to 10% of patients in the placebo arm. Pregabalin 300 mg daily in addition to speech pathology therapy (SPT) was also studied in a similar population of patients suffering from chronic cough (13). In a randomized trial in 40 patients, suppression of cough was found to be better with the addition of pregabalin to SPT as compared to a placebo; the difference in the LCQ score was 3.50; 95% CI, 1.1-5.8; P=0.024 (13). Although the two drugs, pregabalin and gabapentin, have not directly been compared, it seems that at effective doses pregabalin may have resulted in better improvement in cough related QoL (13). An interesting point to note is that the positive effects of pregabalin continued well after stopping the drug though the same was not observed for gabapentin (12,13).

Despite the use of adequate antitussive medications and appropriate doses of opioids, cough remains ‘very distressing’ in 22% patients (14). Given the lacunae in evidence-based management of cough there is a need for newer effective antitussive treatments in lung cancer patients. We propose a study evaluating the role of pregabalin as an antitussive therapy in treating chronic cough in patients with lung cancer.

# AIMS and OBJECTIVES

## Study Hypothesis

The use of the neuromodulatory drug, pregabalin, will reduce cough severity in patients with lung cancer.

## Aim

## To evaluate the efficacy and safety of pregabalin in decreasing the severity of cough in patients with lung cancer compared to placebo.

# Objective

### Primary objective

To study the change in Visual Analog Scale (VAS) scores between baseline and week 9 of treatment with pregabalin (at maximal tolerated dose of 300 mg per day) as compared to placebo.

### Secondary objectives

1. To study the change in Manchester Cough in Lung Cancer Scale (MCLCS) scores between baseline and week 9 of treatment with pregabalin (at maximal tolerated dose of 300 mg per day) as compared to placebo.
2. To study the change in VAS and MCLCS scores between baseline and day 7 of treatment with pregabalin (at maximal tolerated dose of 300 mg per day) compared to placebo.
3. To study the effect of pregabalin (at maximal tolerated dose of 300 mg per day) versus placebo on improvement in quality of life (QOL) in lung cancer patients with chronic cough using EORTC QOL questionnaires.
4. To describe the adverse effects of pregabalin in lung cancer patients with chronic cough.

# Study Methodology

### **3.1 Study Population and setting:**

Patients with suspected/diagnosed lung cancer complaining of cough lasting 2 weeks or longer despite the use of standard cough therapy will be screened for the study. The study will be conducted in the department of medical oncology at Tata Memorial Center located in Mumbai, Maharashtra.

### **3.2 Inclusion criteria**

1. Age 18 years or older.
2. Locally advanced or metastatic suspected or diagnosed NSCLC.
3. Eastern Cooperative Group performance status between 0 and 2.
4. Moderate (grade 2) or severe (grade 3) cough that has lasted for 2 or more weeks. Cough severity will be according to the Common Terminology Criteria for Adverse Events (CTCAE), v.5, in which grade 2 signifies moderate symptoms, medical intervention required, limiting instrumental activities of daily living; and grade 3 cough signifies severe symptoms limiting self-care activities of daily living.
5. Willing and able to limit to one alcoholic beverage per day (e.g. 360 ml of beer or 120 ml of wine or 330 ml of cooler or 43 ml of hard liquor like whisky/vodka/rum/gin).
6. Creatinine clearance measured by the Cockcroft-Gault formula > 60 ml/min.
7. Women of childbearing potential must be willing to consent to using effective contraception (e.g., hormonal contraceptives, bilateral tubal ligation, barrier with spermicide, intrauterine device) while on treatment and for at least 3 months thereafter. A man who is the partner of a woman of childbearing potential must be willing to consent to using effective contraception (e.g., vasectomy or barrier with spermicide) while on treatment and for 3 months thereafter.
8. Capable of providing informed consent and follow trial procedures.

### **3.3 Exclusion criteria**

1. Patients already receiving pregabalin or gabapentin, irrespective of indication, at the time of screening.
2. Known hypersensitivity to pregabalin or gabapentin or their components.
3. Pregnancy or breast feeding.

### **3.4 Study design:**

The study is designed as a randomized double-blind placebo-controlled trial. All patients will sign a written informed consent. Randomization will be done using computer generated blocks. There will be no stratification factors. An independent biostatistician will perform the randomization. Pregabalin and matching placebo will be dispensed to the patient by a designated person, who is not a member of the study team. The investigators, research staff and the patients will be masked to the study arm and will have no access to the randomization schedule. Patients will be randomized to one of the following 2 arms:

Arm A: Pregabalin starting at 75 mg orally daily, with dose escalation over 7 days (according to dose escalation schedule) to a maximum dose of 300 mg orally daily for 9 weeks followed by de-escalation over 7 days, then stop; or

Arm B: Placebo, with dose escalation over 7 days (according to dose escalation schedule), followed by the maximal dose for 9 weeks followed by de-escalation over 7 days, then stop.

### **3.5 Dose escalation schedule:**

Pregabalin or placebo will be started from the day of randomization and the dose will be escalated provided the patient is not experiencing excess toxicity as per the dose escalation schedule described in the table given below. Once dose escalation is completed, pregabalin 300 mg or placebo orally daily will be continued until 9 weeks, i.e. until the first restaging imaging study is performed. After efficacy assessment is completed, pregabalin or the placebo will be tapered (again according to the protocol) over 6 days and then discontinued.

| **Phase** | **Days** | **Daily dose (mg)** | **Schedule (1 capsule=75 mg)** |
| --- | --- | --- | --- |
| Escalation | 1-2 | 75 | 1 capsule, morning |
|  | 3-4 | 150 | 1 capsule, morning and evening |
|  | 5-6 | 225 | 1 capsule, morning, midday and evening |
| Maximal treatment | 7 days to 9 weeks | 300 | 1 capsule, morning and midday;  2 capsules, evening |
| Reduction (start after week 9, once efficacy has been assessed); day numbers counted from the date of assessment of efficacy. | 1-2 | 225 | 1 capsule, morning, midday and evening |
|  | 3-4 | 150 | 1 capsule, morning and evening |
|  | 5-6 | 75 | 1 capsule, morning |
|  | 7 | 0 | Stop |

### 3.6 **Study procedures**

Persons being evaluated for lung cancer in the thoracic medical oncology outpatient department at Tata Memorial Hospital will be questioned about the presence and severity of cough. Patients with cough will be assessed for eligibility. If found eligible and willing to participate in the trial, written informed consent will be obtained. The patient will then be randomized by computer generated blocks to either Arm A (pregabalin 300 mg orally daily) or Arm B (placebo). Investigators, research staff and the patients will be blinded to the study arm. Baseline laboratory tests performed within a week from the date of randomization to assess organ function i.e., complete hemogram, liver and renal function tests and serum electrolytes (sodium, potassium, magnesium, calcium) will be accepted. Evaluation of the performance status and physical examination will be performed by the treating physician. Pulse and blood pressure will be recorded at baseline and every visit.

Information regarding medications that the patient is taking, including additional antitussive medications will be recorded. Plan for oncologic therapy will also be noted.

Tools for assessing cough severity (VAS, MCLCS) will be administered at baseline, on day 7 (+/- 3 days) and 9 weeks (+/- 7 days) from randomization. In patients who are unable to fill out the questionnaires on their own or have trouble reading, the research staff or social workers will help them.

QoL will be assessed using EORCTC QLQ-Q30 and LC13 questionnaires for lung cancer at baseline, day 7 (+/- 3 days) and 9 weeks (+/- 7 days). In patients who are unable to fill out the questionnaires on their own or have trouble reading, the research staff or social workers will help them.

Toxicity data will be recorded and treated as appropriate. The side-effects will be graded according to the Common Terminology Criteria for Adverse Events (CTCAE) v.5. Detailed description of anticipated toxicity and frequency from pregabalin is provided in the next section on adverse events.

- 1. **Adverse events of pregabalin**: In the phase II study evaluating the role of pregabalin and speech pathology combination therapy for patients with chronic refractory cough, the most frequently noted side-effects (all grades) that occurred more commonly with pregabalin as compared to placebo included dizziness (45%), weight gain (25%), blurred vision (20%) and cognitive changes (30%) (13).

As per the product label, the most common adverse events (> 5%) include giddiness, somnolence, dry mouth, peripheral edema, blurred vision, weight gain and cognitive impairment/thinking abnormalities, especially abnormalities with thinking/concentration. Although rare and not reported in the trial in which pregabalin was used for the management of refractory cough, pregabalin may lead to the following severe side-effects

- Angioedema: swelling of the throat, head and neck, which may progress to life-threatening respiratory compromise. Pregabalin should be immediately discontinued.
- Hypersensitivity reactions, like urticaria, wheezing and dyspnea. Pregabalin should be immediately discontinued.
- Increased seizure frequency may occur in patients with seizure disorders, if pregabalin is withdrawn rapidly. Pregabalin must be tapered over 1 week according to the schedule in the protocol
- Pregabalin may cause giddiness and somnolence. Patients should be warned not to drive or operate heavy machinery, while on pregabalin.
  1. **Dose modifications of pregabalin**: Pregabalin is primarily excreted renally. If the patient develops renal impairment (based on the calculated glomerular filtration rate, using the Cockcroft-Gault formula), while on pregabalin, the dose should be modified, based on the calculated creatinine clearance, as follows:

| **Creatinine clearance in ml/min** | **Total pregabalin daily dose (maximal)** |
| --- | --- |
| >60 | 300 |
| 30 to 60 | 150 |
| 15 to 30 | 75 |
| < 15 | 25 – 50 |

In patients who are not tolerating pregabalin due to any intolerable side-effect, the dose of pregabalin should be decreased by 1 step (as per the dose escalation schedule), and this lower dose should be continued for 3 days. If the patient is able to tolerate the lower dose, this should be continued for the duration of the study. If the patient continues to have intolerable side-effects at the lower dose level, the dose of pregabalin may be further reduced, as per the dose escalation table.

Intolerable side-effects of pregabalin will include:

1. Dizziness, grade 3 (severe unsteadiness or sensation of movement; limiting self-care activities of daily living) or any grade but reported intolerable by the patient.
2. Weight gain grade 3 (=/>20% from baseline), which is due to the study drug, and not due to patient responding to cancer-directed therapy with improvement in overall general condition.
3. Blurred vision grade 3 (symptomatic with marked decrease in visual acuity; best corrected visual acuity worse than 20/40 or more than 3 lines of decreased vision from known baseline, up to 20/200; limiting self-care activities of daily living)
4. Cognitive disturbance grade 3 (severe cognitive disability; significant impairment of work/school/life performance)
5. Any other side-effect, which is reported to be intolerable by the patient, and is attributed to the study drug by the investigator.
   1. **Additional medications**: For all patients on either arm, additional cough medications will be added stepwise according the list given below. First, an antitussive should be ordered from the ‘a’ group. In case the patient reports that the cough is not improved after at least 3 days of using the antitussive medication form group ‘1’, this should be stopped and an antitussive from the group ‘2’ should be started, and so on.
6. Expectorant/mucolytic (guaifenesin/bromhexine)
7. Antihistamine (diphenhydramine/chlorpheniramine)
8. Adrenergic blocker (phenylephrine/terbutaline)
9. Narcotic derivative (codeine, dextromethorphan)

Examples of the commercially available cough medications in the various above categories is provided below.

| **Order of prescription** | **Class of antitussive** | **Commercially available cough medications** |
| --- | --- | --- |
| 1 | Expectorant/mucolytic | - Brozedex - Ascoril - Acticuf - Xpect-B |
| 2 | Antihistamine | - Benadryl - Coffdryl - Sinarest - Zedex |
| 3 | Adrenergic blocker | - Terbutaline - Asthalin |
| 4 | Narcotic derivative | - Corex - Alex - Coscopin - Grilinctus |

If patients require other medications while they are on study like corticosteroids (for brain or spinal cord metastases), antibiotics, bronchodilators and opioids (like morphine for pain) these will be permitted, and use will be documented.

- 1. **Cancer-directed therapy**: All patients will be started on cancer-directed therapy, as per the decision of the treating oncologist. The therapeutic regimen with the dose and frequency will be documented. Usually, the first restaging evaluation occurs after 3 cycles of chemotherapy, i.e. after 9 weeks, which will be the time of the end of pregabalin in the cough study.

Although the response of the patients to cancer-directed therapy will impact the cough, we hope that the number of responders to therapy will be roughly equally distributed between the two arms, and that this confounder will be taken care of by means of randomization.

- 1. **Completion of the trial:** The patient will be considered to have completed the trial if:
- Patient has completed the 9-week assessments and has discontinued pregabalin after dose taper
- Patient withdraws consent.
- The investigator feels that it not in the patient’s best interest to continue on the trial.

### **4. Adverse Events**

Information about all adverse events will be collected, recorded and followed up as appropriate. Grading of adverse events will be according to CTCAE v.5. An adverse event is any undesirable sign, symptom or medical condition occurring after starting study treatment, even if the event is not considered to be treatment-related.

Medical conditions/diseases present before starting study treatment will be only considered adverse events if they worsen after starting study treatment. Clinical events occurring before starting study treatment but after signing the informed consent form will be recorded on the Case Report Form. Abnormal laboratory values or test results will constitute adverse events only if they induce clinical signs or symptoms or require therapy and will be recorded on the Adverse Events Case Report Form under the signs, symptoms or diagnosis associated with them.

As far as possible, each adverse event will be described by:

1. its duration (start and end dates)
2. the severity grade- as per CTCAE v.5
3. its relationship to the study drug (suspected /not suspected),
4. the action(s) taken.

**4.1 Serious Adverse Event (SAE)**: Information about all serious adverse events will be collected and recorded on the SAE Report Form and will be reported to the independent ethics committee within 24 hours of learning of its occurrence. An SAE is defined in general as an untoward (unfavorable) event which is fatal or life-threatening, required or prolonged hospitalization, significantly or permanently disabling or incapacitating, may jeopardize the subject and may require medical or surgical intervention to prevent one of the outcomes listed above.

Events not considered to be SAEs are hospitalizations occurring under the following circumstances: were planned before entry into the clinical study; are for elective treatment of a condition unrelated to the studied indication or its treatment; occur on an emergency, outpatient basis and do not result in admission (unless fulfilling the criteria above); are part of the normal treatment or monitoring of the studied indication and not associated with any deterioration in condition.

Any SAE occurring after the patient has provided informed consent and until two weeks after the patient has completed study drug must be reported. This includes the period in which the study protocol interferes with the standard medical treatment given to a patient (e.g. treatment withdrawal during washout period, change in treatment to a fixed dose of concomitant medication). SAEs occurring more than two weeks after study drug discontinuation need only be reported if a relationship to the study drug (or therapy) is suspected.

## ***4.2 Special safety-related procedures/******instructions for rapid notification of SAEs***

To ensure patient safety all SAEs must be reported by the investigator to the Ethics committee within 24 hours of learning of its occurrence, even if it is not felt to be treatment related. Follow-up information about a previously reported SAE must also be reported by the investigators. SAE reports should be forwarded to:

Secretary

Data Safety Monitoring Sub Committee

Tata Memorial Hospital, Mumbai

# **5. Statistical Analysis**

5.1 SAMPLE SIZE CALCULATION: The primary outcome (difference in VAS at baseline and 9 weeks) will used to determine effect size. Sample size will be calculated assuming an effect size of 0.5, with a type I error rate of 5% and power of 80%.

t tests - Means: Difference between two independent means (two groups)

**Input:** Tail(s) = Two

Effect size d = 0.5

α error prob = 0.05

Power (1-β error prob) = 0.80

Allocation ratio N2/N1 = 1

**Output:** Noncentrality parameter δ = 2.828427

Critical t 1.978971

Df = 126

Sample size group A = 64

Sample size group A = 64

Total sample size = 128

Actual power = 0.801460

We will account for a 30% lost-to-follow-up given the fact that a large proportion of patients will have advanced lung cancer. Therefore, the final estimated sample size will be 166.

5.2 STATISTICAL TESTS:

Data will be entered and analyzed using an appropriate statistical program, like SPSS software, version 20 (IBM SPSS Statistics for Windows, Version 20.0. Armonk, NY: IBM Corp.) and R Project for Statistical Computing, version 3.5.1[R Core Team (2013). R: A language and environment for statistical computing. R Foundation for Statistical Computing, Vienna, Austria, URL <http://www.R-project.org/>]*.* Baseline characteristics including demographic data, treatment details, details of the diagnosis and adverse events will be represented as simple percentages.

Statistical Analysis will be done as per intention to treat and per protocol principle. All patients with a complete cough severity assessment at baseline and on 9 weeks will be included for analysis. Patients without any follow-up information after randomization will be excluded. We will use complete-case analysis to handle missing data, i.e. the missing observations will be excluded from the analysis.

To measure cough severity, the VAS score at 9 weeks will be subtracted from the baseline score. The means of the change from the baseline scores will be calculated for all patients in each arm and the means will be compared between the two arms using two-sided Student’s t-test. A *P* value of <0.05 will be considered significant. The effect size will be determined with the use of Cohen’s d statistic, which is a measure of the difference between two means divided by an estimate of a pooled standard deviation. As per conventional classification, an effect size of 0.2 will be considered small, 0.5 moderate, and 0.8 large (19). A similar methodology will be used to evaluate the cough impact using the MCLCS. The change in the VAS scores and MCLCS scores from baseline to day 7 to 9 weeks and will be explored graphically between the arms.

QoL data will be scored as per the procedure described in the EORTC scoring manual (15,16). Different subdomain wise QoL data will be generated from the QoL dataset. A subdomain wise comparison was performed between the arms, using the linear mixed effect model.

The difference in toxicities between the two arms will be compared using the Pearson chi-square test or Fisher Exact test.

**6. Feasibility of the Study:** We see approximately 1300 lung cancer patients in a year at Tata Memorial Hospital. We expect to complete the study within 24 months of approval of study.

# 7. Ethical Considerations

The Investigator or a person designated by him/her will collect informed consent from all participants, prior to which the Investigator or co-investigator must inform each participant of the objectives, benefits, risks and requirements of the study. He/she will also provide the participant with an information sheet in clear, simple language. The study participant will be allowed ample time to inquire about details of the study and to decide whether or not to participate in the study. The study will not commence until approval has been obtained from the Tata Memorial Hospital Human Ethics Committee. The trial will be conducted according to the principles laid down by the International Conference on Harmonization Good Clinical Practice guidelines, the Declaration of Helsinki, Schedule Y (Drugs and Cosmetic Act 1940) and the guidelines established by the Indian Council of Medical Research (ICMR).

# Appendix

1. Side effects of pregabalin
2. Visual Analog scale for Cough

.
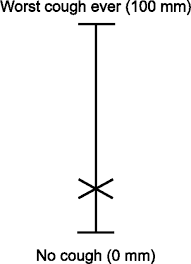


1. Manchester cough in lung cancer scale

# References

1. Wee B. Oxford Textbook of Palliative Medicine. J R Soc Med. 2004 Jul;97(7):356–7.

2. Wong MCS, Lao XQ, Ho K-F, Goggins WB, Tse SLA. Incidence and mortality of lung cancer: global trends and association with socioeconomic status. Sci Rep. 2017 Oct 30;7(1):14300.

3. Song W-J, Chang Y-S, Faruqi S, Kang M-K, Kim J-Y, Kang M-G, et al. Defining Chronic Cough: A Systematic Review of the Epidemiological Literature. Allergy Asthma Immunol Res. 2016 Mar;8(2):146–55.

4. Kvale PA. Chronic Cough Due to Lung Tumors. Chest. 2006 Jan;129(1):147S-153S.

5. Harle ASM, Blackhall FH, Smith JA, Molassiotis A. Understanding cough and its management in lung cancer. Curr Opin Support Palliat Care. 2012 Jun;6(2):153.

6. Fields H. State-dependent opioid control of pain. Nat Rev Neurosci. 2004 Jul;5(7):565–75.

7. Vernon M, Kline Leidy N, Nacson A, Nelsen L. Measuring cough severity: development and pilot testing of a new seven-item cough severity patient-reported outcome measure. Ther Adv Respir Dis. 2010 Aug;4(4):199–208.

8. Lövgren M, Tishelman C, Sprangers M, Koyi H, Hamberg K. Symptoms and problems with functioning among women and men with inoperable lung cancer--a longitudinal study. Lung Cancer Amst Neth. 2008 Apr;60(1):113–24.

9. Molassiotis A, Smith JA, Bennett MI, Blackhall F, Taylor D, Zavery B, et al. Clinical expert guidelines for the management of cough in lung cancer: report of a UK task group on cough. Cough Lond Engl. 2010 Oct 6;6:9.

10. Molassiotis A, Bailey C, Caress A, Tan J-Y. Interventions for cough in cancer. Cochrane Database Syst Rev. 2015 May 19;5:CD007881.

11. Yancy WS, McCrory DC, Coeytaux RR, Schmit KM, Kemper AR, Goode A, et al. Efficacy and tolerability of treatments for chronic cough: a systematic review and meta-analysis. Chest. 2013 Dec;144(6):1827–38.

12. Ryan NM, Birring SS, Gibson PG. Gabapentin for refractory chronic cough: a randomised, double-blind, placebo-controlled trial. The Lancet. 2012 Nov;380(9853):1583–9.

13. Vertigan AE, Kapela SL, Ryan NM, Birring SS, McElduff P, Gibson PG. Pregabalin and Speech Pathology Combination Therapy for Refractory Chronic Cough. Chest. 2016 Mar;149(3):639–48.

14. Edmonds P, Karlsen S, Khan S, Addington-Hall J. A comparison of the palliative care needs of patients dying from chronic respiratory diseases and lung cancer. Palliat Med. 2001 Jul;15(4):287–95.

15. Fayers PM, Aaronson NK, Bjordal K, et al, on behalf of the EORTC Quality of Life Group. The EORTC QLQ-C30 Scoring Manual (3rd Edition). Published by: European Organisation for Research and Treatment of Cancer, Brussels 2001.

16. Bergman B, Aaronson NK, Ahmedzai S, et al: The EORTC QLQLC13: a modular supplement to the EORTC Core Quality of Life Questionnaire (QLQ-C30) for use in lung cancer clinical trials. EORTC Study Group on Quality of Life. Eur J Cancer 30A:635-642, 1994

17. Burnham J, Buffin O, Blackhall F*, et al.* P58 The Characterisation and Subjective Assessment of Cough in Lung Cancer and Mesothelioma: The “CLAIM” Study.*Thorax*2013;**68:**A101.

18. Buffin O, Burnham J, Smith J*, et al.P*59 The Characterisation of Cough in Lung Cancer. *Thorax*2013;**68:**A101-A102.

19. Sedgwick P. Randomised controlled trials: understanding effect sizes. BMJ. 2015 Mar 27;350:h1690
